# Atypical Central Serous Chorioretinopathy

**DOI:** 10.4274/tjo.38039

**Published:** 2017-08-15

**Authors:** Zafer Cebeci, Merih Oray, Şerife Bayraktar, İlknur Tuğal-Tutkun, Nur Kır

**Affiliations:** 1 İstanbul University İstanbul Faculty of Medicine, Department of Ophthalmology, İstanbul, Turkey

**Keywords:** central serous chorioretinopathy, Vogt-Koyanagi-Harada disease, Corticosteroid

## Abstract

Bullous central serous chorioretinopathy (CSCR) is a rare variant of CSCR characterized by severe serous retinal detachment which especially involves the inferior quadrants. Corticosteroid therapy administered for systemic or ocular misdiagnoses may induce and exacerbate CSCR. The purpose of this study was to report diagnosis and treatment results of an unusual case of bullous CSCR induced by systemic and periocular corticosteroid therapy received at another medical center due to a misdiagnosis of Vogt-Koyanagi-Harada disease.

## INTRODUCTION

Central serous chorioretinopathy (CSCR) is an idiopathic chorioretinal disease which causes serous detachment in the neurosensory retina, which may also be accompanied by pigment epithelium detachment.^1^ Compared to the acute and chronic types of CSCR, bullous CSCR is rarely reported and is characterized by bullous retinal detachment in the inferior quadrants of the fundus.^2,3,4^ Although its pathophysiology is unknown, corticosteroids are known to be one of the major risk factors in the development of CSCR.^1,5,6,7,8^ The use of corticosteroids, especially in the treatment of systemic or ocular conditions may lead to acute emergence or exacerbation of CSCR.^5,6,7,8,9^ Atypical, bullous, or chronic type CSCR may be confused with other diseases that can cause intraocular inflammation such as Vogt-Koyanagi-Harada (VKH) disease, posterior scleritis, sympathetic ophthalmia, multifocal choroiditis, and serpiginous choroiditis.^5,9^ Corticosteroid treatment due to the misdiagnosis of posterior uveitis leads to the exacerbation of CSCR symptoms.^9^

In the present study, we present the follow-up and treatment response of a patient diagnosed with bullous CSCR who was treated for an extended period with corticosteroids for a diagnosis of VKH.

## CASE REPORT

**Figure 1:**
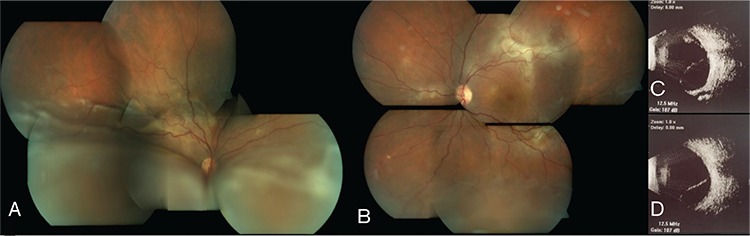
Fundus photography and ultrasonography (USG) images from the patient’s right and left eyes. A) Bullous retinal detachment extending to the superotemporal vascular arcade in the right eye; B) Serous retinal detachment in the inferior periphery and subretinal fibrin visible at the inferior and superior temporal arcades and in the nasal quadrant in the left eye; C) Retinal detachment in the inferior and superior quadrants on the USG in the right eye; D) Retinal detachment in the inferior quadrant on USG in the left eye

**Figure 2:**
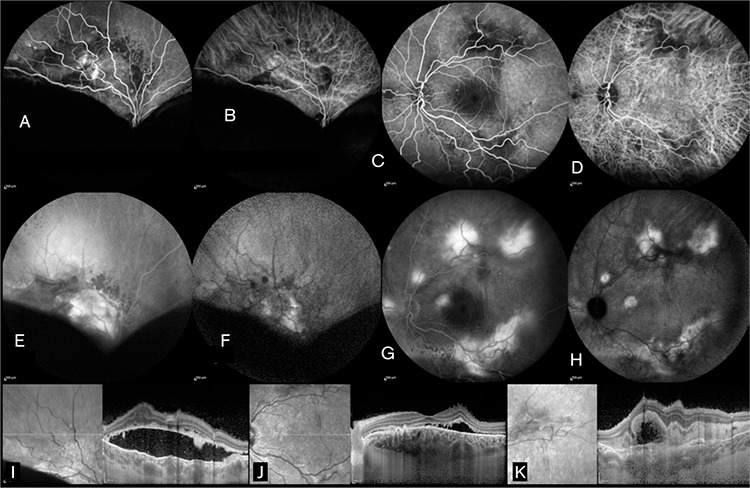
A) Fluorescein angiography (FA) in the right eye revealed an area of early hypofluorescence in the inferior quadrants and hyperfluorescence at the superotemporal vascular arcade; B) Indocyanine green angiography (ICGA) in the right eye revealed early hypofluorescence in the inferior quadrants and dilated choroidal vessels at the superotemporal arcade; C) FA in the left eye revealed early hypofluorescence at the superior and inferior temporal arcades and in the nasal quadrant; D) ICGA in the left eye revealed dilated choroidal vessels at the level of the temporal vascular arcade; E) FA in the right eye showed hyperfluorescence at the superotemporal vascular arcade due to leakage and hypofluorescence in the inferior quadrants at late phases; F) ICGA in the right eye showed late hyperfluorescence at the superotemporal arcade and hypofluorescence in the inferior quadrants; G) left eye revealed increased hyperfluorescence in late phases in the macula, superotemporal and inferotemporal arcades, and nasal quadrant; H) ICGA in the left eye showed hyperfluorescence in the macula, superotemporal and inferotemporal vascular arcades, and nasal quadrant in late phases; I) In the right eye, subretinal fluid and a subretinal hyperreflective band are visible on enhanced depth imaging-optic coherence tomography (EDI-OCT) from the section passing through the superotemporal vascular arcade; J) In the left eye, the macular EDI-OCT section reveals subretinal fluid, retinal pigment epithelium irregularities, internal limiting membrane folds, and a subfoveal choroid thickness of 591 µm; K) In the left eye, the OCT cross-section taken at the level of the superotemporal arcade reveals subretinal accumulation of hyperreflective material and a hyporeflective space

**Figure 3:**
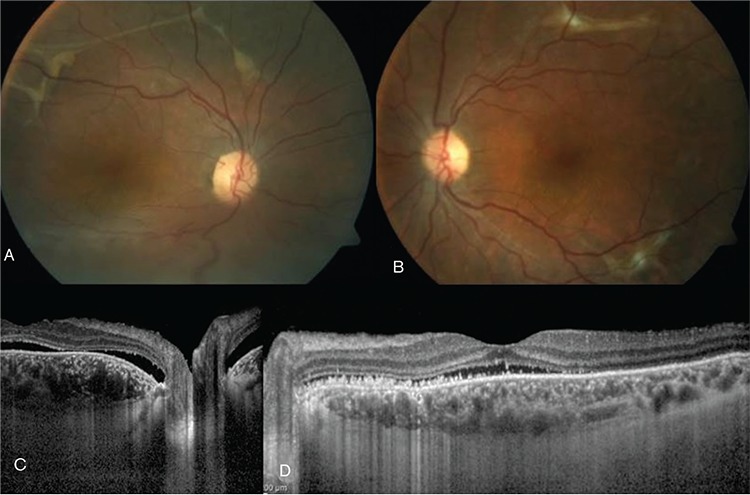
Fundus images from the left and right eyes taken 4 months after treatment. A) In the right eye, serous retinal detachment regressed to the level of the inferotemporal arcade, while a subretinal band is apparent at the level of the superotemporal arcade; B) In the left eye, subretinal fibrosis is apparent at the superior and inferior temporal arcades; C) Enhanced depth imaging-optic coherence tomography (EDI-OCT) in the right eye shows subretinal fluid and subfoveal choroid thickness of 537 µm in the section taken at the macula; D) EDI-OCT in the left eye revealed subretinal fluid in the macula, hyperreflective material in subretinal area, and subfoveal choroid thickness of 335 µm

A 28-year-old female patient presented to our clinic for progressive decrease in vision, first the right eye and later the left eye, for the past year. Her medical history was unremarkable in terms of systemic disease. She reported going to another center one year earlier for reduced vision where she was diagnosed with VKH and treated 3 times with high-dose corticosteroid and finally with an injection in her right eye. At time of presentation, the patient was using oral methylprednisolone 16 mg/day, oral cyclosporine 150 mg/day, and oral azathioprine 75 mg/day. Physically, she exhibited cushingoid appearance and complained of excessive weight gain and hair growth on the body. On examination, her visual acuity was light perception with projection in the right eye and 0.6 in the left eye. Anterior segment examination of both eyes was normal except for white accumulations consistent with corticosteroid in the inferior subconjunctival region of the right eye. Intraocular pressure measurement was 13 mmHg in the right and 15 mmHg in the left eye with dorzolamide hydrochloride-timolol maleate twice daily. Fundoscopic examination revealed no cells in the vitreous and exudative retinal detachment from the inferior quadrants to the superotemporal arcade in the right eye. In the left eye, there were no cells in the vitreous, while exudative retinal detachment was observed in the inferior periphery and subretinal fibrin accumulation was noted at the superotemporal and inferotemporal arcades and nasal to the optic disc (Figure 1A, B). Ultrasonographic imaging (USG) was compatible with exudative retinal detachment in the right and left eyes (Figure 1C, D). Fluorescein angiography (FA) and indocyanine green angiography (ICGA) in the right eye revealed hypofluorescence in the region corresponding to the exudative retina detachment, as well as early hyperfluorescence increasing in later phases at the peripheral superotemporal arcade surrounded by multiple localized hypofluorescent foci. In the left eye, FA and ICGA revealed early hyperfluorescence increasing in later phases in the macula, superior and inferior temporal arcades, and nasal of the optic nerve (Figure 2A-H). Enhanced depth imaging-optical coherence tomography (EDI-OCT) revealed subretinal fluid and a hyperreflective band located subretinally in the section taken at the peripheral superotemporal arcade in the right eye; in the left eye, EDI-OCT showed subretinal fluid on the section passing through the macula, and subretinal hyperreflective material with a hyporeflective field and irregularity in retinal pigment epithelium (RPE) in the section taken at the level of the superotemporal arcade (Figure 2I-K). Subfoveal choroidal thickness in the left eye was determined as 591 µm on EDI-OCT (Figure 2J). Neurologic and otorhinolaryngologic examinations were normal; however, dermatologic examination revealed findings consistent with hirsutism. Based on the results of ophthalmologic examination and auxiliary imaging techniques, the patient was diagnosed with bullous type CSCR exacerbated by corticosteroid therapy. After consultation with endocrinologists, the patient was diagnosed with Cushing’s syndrome associated with systemic corticosteroid use, and gradual methylprednisolone tapering was recommended. Therapy with cyclosporine and azathioprine was discontinued. The subconjunctival corticosteroid particles were removed from the patient’s right eye. After consultation with endocrinology and obtaining the patient’s consent, treatment was initiated with oral mineralocorticoid receptor antagonist eplerenone 25 mg twice daily. Low fluence photodynamic therapy (PDT) (25 J/cm^2^, 300 mW/cm^2^) was applied to areas of leakage seen on FA and ICGA in the macula and superotemporal arcade in the left eye due to the potential threat to the macula. Focal laser photocoagulation was applied to areas of leakage in the left nasal and inferotemporal arcades. Exudative detachment was reduced in the right eye and had completely regressed in the left eye at 1-month follow-up. At month 4, visual acuity was counting fingers from 1 meter in the right eye and had improved to 0.7 in the left eye. Anterior segment examination was normal in both eyes. Fundus examination in the right eye revealed regression of the exudative detachment to the inferotemporal arcade and the presence of subretinal fibrosis at the peripheral superotemporal arcade; in the left eye, subretinal fibrosis was observed at the inferior and superior temporal arcades (Figure 3A, B). EDI-OCT examination revealed subretinal fluid in the right and left maculas, and subfoveal choroidal thickness was 537 µm in the right eye and 335 µm in the left eye (Figure 3C, D). The patient was lost to follow-up after the fourth month because she moved to another country.

## DISCUSSION

Although it is not fully understood how corticosteroids induce or exacerbate CSCR, various mechanisms have been implicated in the development of the disease. Exogenous or endogenous hypercortisolemia elevates catecholamine levels, creates a mineralocorticoid effect, or induces thrombocyte aggregation, which increase choriocapillaris permeability and RPE decompensation, leading to CSCR.^1,9^ The development of CSCR is independent of the corticosteroid type, dose or route of administration.^9^ Gass and Little^5^ reported bilateral bullous CSCR in a patient who was administered systemic and sub-Tenon corticosteroid due to a misdiagnosis of choroiditis; the serous detachments regressed after discontinuing the drug and treating with laser photocoagulation. In a series reporting the long-term follow-up outcomes of 25 patients with bullous CSCR, 4 patients (16%) developed this severe form of CSCR in which the areas of serous detachment healed with residual scarring or atrophy after drug discontinuation and treatment.^4^

The findings of bullous CSCR may be confused with uveal effusion, metastatic carcinoma or lymphoma, rhegmatogenous retinal detachment, and diseases that cause inflammation such as VKH disease, multifocal choroiditis, and sympathetic ophthalmia, and misdiagnosis results in unnecessary tests and treatments.^5,9^ Kang et al.^10^ observed progression of bullous detachment in a 47-year-old male patient who was treated with systemic corticosteroids for a prediagnosis of VKH; they subsequently discontinued the medication and successfully treated the patient with vitrectomy and internal subretinal fluid drainage. Gao and Li^11^ reported a patient with a previous history of CSCR whose disease converted to the bullous form after being treated with systemic methylprednisolone due to misdiagnosis of VKH. A multimodal imaging approach utilizing FA, ICGA, and spectral domain-OCT is important in the differential diagnosis and follow-up of the disease.^12^ CSCR is most commonly mistaken for the exudative retinal detachment seen in the acute phase of VKH. Findings that facilitate the diagnosis of CSCR are an absence of cells in the anterior chamber or vitreous and no sign of optic disc edema on examination; no choroidal thickening in USG; an absence of optic disc staining in late phases of FA; and observing multifocal hyperpermeability instead of diffuse hyperpermeability and the absence of hypofluorescence spots or optic disc staining on ICGA.9 Other findings that support CSCR diagnosis are the presence of dome-shaped serous detachment on OCT, subretinal precipitates, localized fibrin reaction, presence of RPE detachment and irregularities, no visible subretinal septa, fundus autofluorescence showing hypoautofluorescence in the area of subretinal fluid, and hyperautofluorescence corresponding with areas of leakage on FA.^9^ Because choroid thickness increases in both VKH disease and CSCR, noninvasive EDI-OCT examination is not useful for differentiating between these two entities.^13^ However, corticosteroid treatment provides favorable results in VKH ocular involvement, but may exacerbate ocular symptoms in CSCR.^9^ In our study, our patient also received an initial diagnosis of VKH due to the presence of presumed serous retinal detachment, and was administered systemic and periocular corticosteroid therapy for one year. These treatments not only exacerbated the CSCR and induced its transformation to the bullous form, but also caused systemic problems such as Cushing’s syndrome.

Due to its angio-occlusive effect, PDT leads to narrowing of the choroidal vessels and thereby a reduction in choroidal exudation, as well as reshaping of choroidal vessels.^14^ Low-fluence or low-dose PDT is used in CSCR to avoid the potential complications of standard PDT, such as RPE atrophy, choroidal ischemia, and secondary choroidal neovascularization. Successful outcomes have been reported in studies using these techniques.^15,16^ Although there are no randomized, controlled studies in the literature, some studies have shown that oral eplerenone, a mineralocorticoid receptor antagonist, decreases subretinal fluid in chronic CSCR and is a promising treatment method for the future.^17,18^ Because our patient had very advanced stage disease, we combined available treatment methods such as low-fluence PDT, focal laser photocoagulation, and oral eplerenone, and the patient responded well within a short period.

In conclusion, bullous type CSCR may be confused with ocular symptoms of acute VKH disease. Corticosteroid therapy administered for a misdiagnosis of intraocular inflammation may exacerbate CSCR and lead to irreversible damage. Atypical bullous CSCR must be considered in cases of serous retinal detachment, and the use of multimodal imaging methods in addition to detailed ophthalmologic and systemic examinations will facilitate an accurate diagnosis before giving corticosteroids.
